# Attentional Control of Gait and Falls: Is Cholinergic Dysfunction a Common Substrate in the Elderly and Parkinson’s Disease?

**DOI:** 10.3389/fnagi.2016.00104

**Published:** 2016-05-09

**Authors:** Elisa Pelosin, Carla Ogliastro, Giovanna Lagravinese, Gaia Bonassi, Anat Mirelman, Jeffrey M. Hausdorff, Giovanni Abbruzzese, Laura Avanzino

**Affiliations:** ^1^Department of Neuroscience, Rehabilitation, Ophthalmology, Genetics and Maternal Child Health, University of GenoaGenoa, Italy; ^2^Department of Experimental Medicine, Section of Human Physiology and Centro Polifunzionale di Scienze Motorie, University of GenoaGenoa, Italy; ^3^Center for the Study of Movement, Cognition and Mobility, Department of Neurology, Tel Aviv Sourasky Medical CenterTel Aviv, Israel; ^4^Department of Physical Therapy, Sackler Faculty of Medicine and Sagol School of Neuroscience, Tel Aviv UniversityTel Aviv, Israel

**Keywords:** Parkinson’s disease, dual task gait, falls, transcranial magnetic stimulation (TMS), cholinergic system, attention

## Abstract

The aim of this study was to address whether deficits in the central cholinergic activity may contribute to the increased difficulty to allocate attention during gait in the elderly with heightened risk of falls. We recruited 50 participants with a history of two or more falls (33 patients with Parkinson’s Disease and 17 older adults) and 14 non-fallers age-matched adults. Cholinergic activity was estimated by means of short latency afferent inhibition (SAI), a transcranial magnetic stimulation (TMS) technique that assesses an inhibitory circuit in the sensorimotor cortex and is regarded as a global marker of cholinergic function in the brain. Increased difficulty to allocate attention during gait was evaluated by measuring gait performance under single and dual-task conditions. Global cognition was also assessed. Results showed that SAI was reduced in patients with PD than in the older adults (fallers and non-fallers) and in older adults fallers with respect to non-fallers. Reduction in SAI indicates less inhibition i.e., less cholinergic activity. Gait speed was reduced in the dual task gait compared to normal gait only in our faller population and changes in gait speed under dual task significantly correlated with the mean value of SAI. This association remained significant after adjusting for cognitive status. These findings suggest that central cholinergic activity may be a predictor of change in gait characteristics under dual tasking in older adults and PD fallers independently of cognitive status.

## Introduction

Falls are a major cause of morbidity among older adults and individuals with neurodegenerative diseases, such as Parkinson’s disease (PD). One potential mechanism underlying the increased fall risk in those populations is reduced attentional resource allocation, which can compromise postural and gait stability (Montero-Odasso et al., 2012). Although walking has long been considered a primarily automatic motor task, emerging evidence suggests that this view is overly simplistic (Hausdorff et al., 2005; Amboni et al., 2013). Almost 20 years ago, the seminal “stops walking while talking” study by Lundin-Olsson et al. (1997) showed that the inability to maintain a conversation while walking is a marker of future falls in older nursing home residents. Since then, many studies have used the “dual-task paradigm” (i.e., observing people walking while they perform a secondary attention-demanding task) to assess the interactions between cognition (specifically reduced capacity to sustain and divide attention), gait and propensity to fall. It is well known that, the cholinergic system plays a vital role in the top-down control of attentional orienting and stimulus discrimination (Klinkenberg et al., 2011). Thus, a dysfunction of the cholinergic system has been identified as potentially contributing to the increased fall propensity and reduced attentional capacity in aging and neurodegeneration. Recent imaging data support the relationship between central cholinergic activity and falls and slower gait speed in patients with PD (Bohnen et al., 2009, 2013). Further, an association between cholinergic dysfunction and gait disturbances in older adults with moderate to severe cognitive impairment has been reported (Morris et al., 1987; Montero-Odasso et al., 2015). Recent pilot work intriguingly suggested that treatment with anti-cholinergic medications may the risk of falls (Mancini et al., 2015; Henderson et al., 2016).

To date, however, it is not clear if cholinergic activity is abnormal in PD fallers or elderly fallers, without clinical diagnosis of dementia or other severe cognitive impairment, and if this contributes to dual task gait disturbances, a marker of fall risk.

To this aim, we assessed central cholinergic activity by means of short latency afferent inhibition (SAI), a transcranial magnetic stimulation (TMS) technique (Tokimura et al., 2000) in two groups of “fallers” (older adults and patients with PD), and a control group of older adults non-fallers. SAI is thought to be related to central cholinergic activity, given that in normal subjects it can be reduced or abolished by muscarinic antagonist scopolamine (Di Lazzaro et al., 2000). To explore the possible contribution of cholinergic activity to gait dysfunction, we assessed gait speed under single and dual-task conditions.

## Materials and Methods

### Participants

This cross-sectional study involved 64 participants recruited at the Department of Neuroscience, University of Genoa. Particularly, this study involved 50 participants recruited for the V-TIME study and 14 additional participants (Mirelman et al., 2013).

Three age-matched groups of participants were included: (i) 33 PD fallers (PD-F, 17 males and 16 females); (ii) 17 older adults fallers (OLD-F, 7 males and 10 females); and (iii) 14 older adults non-fallers (OLD-NF, 9 males and 5 females; Table 1). Participants were considered as “fallers” if they reported two or more falls within the 6 months prior to the beginning of the study. Common inclusion criteria were: (i) age between 60 and 85 years and (ii) able to walk for 5 min unassisted. Patients with PD were included if they had idiopathic PD, as defined by the UK Brain Bank criteria, were in Hoehn and Yahr stage II-III, and were medically stable for at least 1 month prior to the study. Common exclusion criteria were: (i) a clinical diagnosis of dementia or other severe cognitive impairment (MMSE <24); (ii) psychiatric co-morbidity; and (iii) history of stroke or other neurologic disorders. All evaluations were performed in the morning approximately 90 min after the first dopaminergic drugs intake or when PD patients were in their best “on” medication state. The experimental protocol was approved by the Ethics committee of the University of Genoa (141/12). Prior to any procedures, the study was fully explained to the participants and written informed consent was obtained from all subjects.

**Table 1:** **Demographics and clinical characteristics of participants**.

	PD patients fallers (mean ± SD)	Older adults fallers (mean ± SD)	Older adults non-fallers (mean ± SD)	*P*	*Post hoc* (Mann Whitney)
Age (years)	72.6 ± 4.4	73.4 ± 4.2	72.1 ± 4.9	>0.05	–
Male/Female	17/16	7/10	9/5	>0.05	
Education (years)	9.8 ± 4.6	11.2 ± 4.4	10.8 ± 4.1	>0.05	–
UPDRS III (score)	30.3 ± 9.13	–	–	–	–
LEDD (mg)	775.5 ± 358.6	–	–	–	–
Falls in 6 months prior to study (number)	3.8 ± 2.1	2.6 ± 1.0	–	0.031	–
MoCA (score)	23.7 ± 3.9	26.4 ± 1.6	28.3 ± 2.0	<0.000	^§^*p* = 0.006
					^#^*p* < 0.000
					^∞^*p* = 0.012

*PD-F, Parkinson’s Disease fallers; OLD-F, Older adults fallers; OLD-NF, Older adults non fallers; UPDRS, Unified Parkinson’s Disease Rating Scale; LEDD, Levodopa Equivalent Daily Dose; MoCA, Montreal Cognitive Assessment; *Post hoc*: ^§^PD-F vs. OLD-F; ^#^PD-F vs. OLD-NF; ^∞^OLD-F vs. OLD-NF*.

### Assessment

For PD patients, disease severity was evaluated using the Movement Disorders Society (MDS) Unified Prkinson’s Disease Rating Scale (UPDRS) section III (Motor Examination) and levodopa (L-DOPA) equivalent dose was calculated for each patient. In all participants, global cognitive function was evaluated with the Montreal Cognitive Assessment (MoCA).

Participants were asked to walk in a well-lit corridor under two conditions, each lasting 1 min: (i) walking at a comfortable speed, (ii) walking during a verbal fluency task. The GaitRite mat, a sensorized 7-meter carpet (CIR Systems, Inc. Haverton, MA, USA), captured individual footfall data using embedded pressure sensors. The ProtoKinetics Movement Analysis Software was used to analyze the data. Gait speed was determined.

### Short Latency Afferent Inhibition (SAI)

Subjects were seated in a comfortable chair with a mounted headrest during the experiments. Electromyography was recorded with silver disc surface electrodes placed on a tendon belly arrangement over the bulk of the first dorsal interosseus muscle and the first metacarpophalangeal joint bilaterally. Electromyography signals were amplified and filtered (20 Hz to 1 kHz) with a D360 amplifier (Digitimer). The signals were sampled at 5000 Hz, digitized using a laboratory interface (Power1401; Cambridge Electronics Design), and stored on a personal computer for display and later offline data analysis.

TMS was performed with a figure-of-eight coil connected to a Magstim 200^2^ magnetic stimulator (Magstim, UK). The coil was placed tangentially to the scalp with the handle pointing backwards and laterally at a 45° angle to the sagittal plane inducing a posterior–anterior current in the brain. We determined the “motor hot spot” for activation of the first digital interosseeus. At the beginning of each experiment, the stimulus intensity needed to evoke motor evoked potentials (MEPs) of approximately 0.8–1.0 mV peak-to-peak amplitude was defined. MEPs were recorded from the more affected side in PD patients and from the dominant side in the older adults.

SAI was tested with a suprathreshold test TMS stimulus over the hand area, adjusted to produce MEPs of 1 mV in amplitude, preceded by an electrical conditioning stimulus over the contralateral median nerve at the wrist (cathode proximal, constant square wave current, duration 200 microseconds (200 μs), intensity set just above threshold for evoking a small twitch in the opponens pollicis muscle). SAI was randomly tested at six different inter-stimulus intervals, with 15 trials at each inter-stimulus interval (conditioned trials: at 18, 20, 22, 24, 26 and 28 ms) and with 20 unconditioned (test) stimuli also delivered randomly.

### Data Analyses

Regarding gait parameters, gait speed was calculated under normal and dual task gait and expressed in meters/seconds (m/s). To evaluate changes in gait speed induced by dual task with respect to single task, a delta value was calculated as follows: (gait speed_dual task−_gait speed_single task_)/gait speed_single task_) × 100. For TMS, the amplitude of the conditioned responses was expressed as a percentage of the amplitude of the test response. We averaged the percentage of inhibition of the conditioned responses at the six different inter-stimulus intervals to obtain a grand mean of SAI.

### Statistical Analyses

Gender differences between the groups were assessed by Chi-square test. We checked that data were normally distributed (*Shapiro-Wilk W test*). Differences between groups for age, education and MoCA were assessed by the non-parametric Kruskall-Wallis test and *post hoc* comparisons were done with the Mann-Whitney *U*-test. Differences between OLD-F and PD-F for number of falls over the past 6 months were assessed by means of an unpaired *t*-test. Gait speed under single and dual-task conditions were entered in a repeated measures ANOVA (RM-ANOVA) with the factor GROUP as between subjects factor and the factor CONDITION (normal and dual-task gait) as the within subjects factor. Grand mean of SAI was compared groups by means of a one-way ANOVA. The correlation between dual task costs for gait speed and the grand mean of SAI was evaluated by means of Spearman correlation analysis. The level of significance was set at *p* < 0.05, *post hoc* analysis was performed using *t* tests, applying the Bonferroni correction for multiple comparisons where appropriate. To assess whether cortical cholinergic activity was an independent predictor of change in gait characteristics under dual task condition, a stepwise linear regression model was constructed adjusted for age and MoCA score. A standard statistical package computed odds ratios (ORs), two-sided 95% confidence intervals (CIs) and *p* values; *p* < 0.05 was considered to be significant. All statistical analyses were performed using SPSS22.

## Results

The demographic, clinical and cognitive data are summarized in Table 1. There were no significant differences between age, gender and education levels in the three groups (*p* > 0.05). Number of falls over the past 6 months was significantly higher in PD-F than in OLD-F (*p* = 0.03). For MoCA there was a significant difference among the groups (*p* < 0.001). *Post hoc* analysis revealed a significant difference between PD-F and OLD-F with respect to OLD-NF and also between PD-F and OLD-F (Table 1).

**Figure 1:**
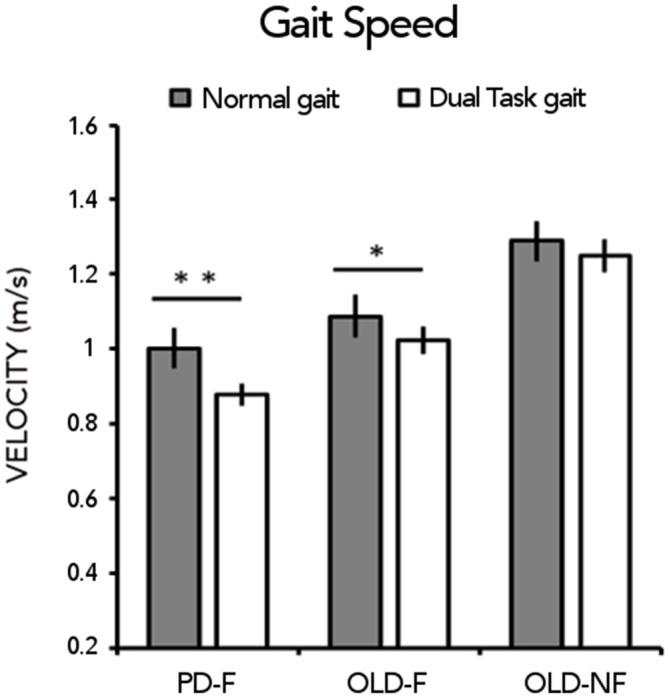
**Gait speed (m/s) under normal gait and dual task gait in the three groups of subjects (Parkinson’s Disease fallers, PD-F; Older adults fallers, OLD-F; Older adults non-fallers, OLD-NF).** Asterisks indicate statistical significant difference within group between normal and dual task gait (*p < 0.05, **p < 0.01).

For gait speed (Figure 1), RM-ANOVA showed a significant main effect of CONDITION (*F*_(1,61)_ = 25.89; *p* < 0.001) and GROUP (*F*_(2,61)_ = 14.91;*p* < 0.001) and a significant GROUP × CONDITION interaction (*F*_(2,61)_ = 3, 29; *p* = 0.04). During usual walking, gait speed was significantly lower in PD-F than in OLD-NF (*p* < 0.001) and in OLD-F than in OLD-NF (*p* = 0.003) with no difference between PD-F and OLD-F (*p* = 0.12). During dual task walking, gait speed was significantly lower in PD-F than in both OLD-F (*p* = 0.025) and OLD-NF (*p* < 0.001) and in OLD-F than in OLD-NF (*p* = 0.005). Consistent with this, PD-F and OLD-F significantly reduced their gait speed under dual task gait with respect to normal gait (PD-F, dual vs. normal *p* < 0.001; OLD-F, dual vs. normal *p* = 0.022), whereas OLD-NF did not significantly reduced gait speed under dual task gait (*p* = 0.18).

**Figure 2:**
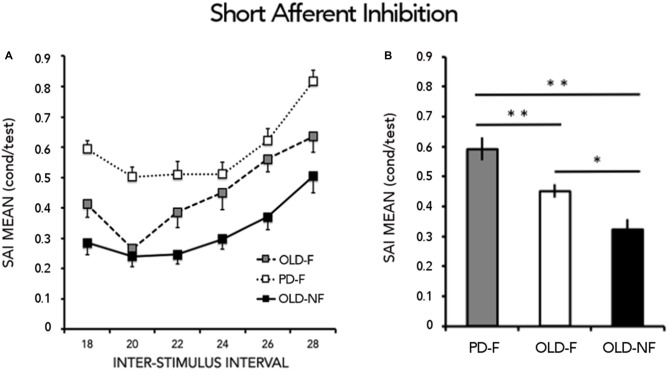
**Results from short latency afferent inhibition (SAI). (A)** Time course of inhibition. Abscissa indicates the interstimulus intervals (ISIs) whereas ordinate indicates the size of the conditioned response, expressed as a percentage of the unconditioned motor evoked potentials (MEPs). **(B)** Grand mean of SAI calculated by combining across all interstimulus intervals (ISIs) the conditioned responses and expressed as the percentage of the unconditioned MEPs. Asterisks indicate significant between groups difference (*p < 0.05, **p < 0.01). PD-F, Parkinson’s Disease fallers; OLD-F, Older adults fallers; OLD-NF, Older adults non fallers.

**Figure 3:**
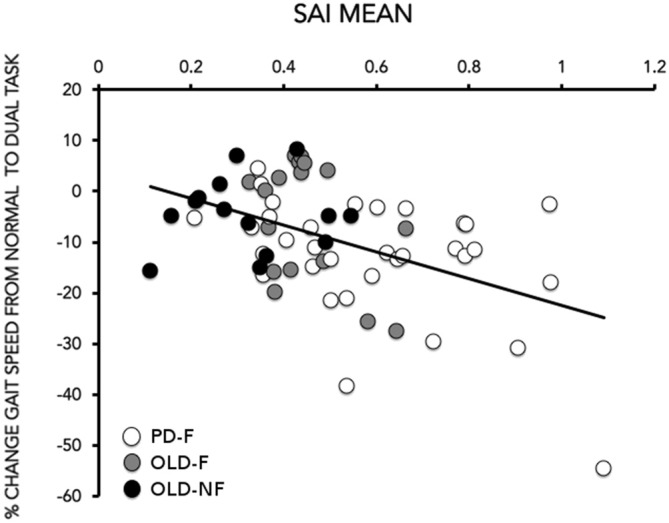
**Correlation between the grand mean of SAI (*X*-axis) and the % change in gait speed (gait speed) from normal to dual task (*Y*-axis).** The % change in gait speed was calculated as (gait speed_dual task−_gait speed_single task_)/gait speed_single task_) × 100. Data of PD (white circles), older adults fallers (OLD-F: gray circles) and older adults non-fallers (OLD-NF: black circles) are plotted together.

SAI results are reported in Figure 2A. One way-ANOVA showed a significant effect of GROUP (*F*_(2,61)_ = 12.15; *p* < 0.001), There was significant less SAI in PD-F than in OLD-F (*p* = 0.010) and OLD-NF (*p* < 0.001), and in OLD-F than in OLD-NF (*p* = 0.045; Figure 2B). Further, the dual task change in gait speed was significantly correlated with the grand mean of SAI in our population (Rho = −0.39, *p* = 0.001; Figure 3); subjects who had lower cortical cholinergic activity, tended to have a greater decrease in gait speed during the dual task gait.

Univariate linear regression analysis showed that the significant association between the grand mean of SAI and gait speed change induced by dual task condition remained significant after adjusting for age and MoCA score (Table 2).

**Table 2:** **Regression model statistics and coefficients of variables for dual task cost for walking speed (PD, OLD-F and OLD-NF)**.

Gait speed change DT	*P*	Stand. β	*R*^2^	95% CI
SAI mean	<0.001	−0.46	0.22	[−39.0, −13.7]
Adjusted for age	<0.001	−0.47	0.23	[−39.6, −14.3]
Adjusted for age, MoCA	0.015	−0.34	0.27	[−34.8, −3.9]

*PD-F, Parkinson’s Disease fallers; OLD-F, Older adults fallers; OLD-NF, Older adults non-fallers; SAI, short latency afferent inhibition; DT, dual task; MoCA, Montreal Cognitive Assessment*.

## Discussion

The main results of the present study were the following: First, we found that our faller population manifested a cholinergic dysfunction with respect to older adults non-fallers. Second, change in gait speed in the dual task condition significantly correlated with cholinergic dysfunction, suggesting that subjects with lower central cholinergic activity were more likely to have a larger decrease in gait speed induced by dual task. Finally, we found that central cholinergic activity was an independent predictor of change in gait characteristics under dual task in our faller population regardless of cognitive status even if, as expectable, older adults performed better than PD on MoCA.

Strong evidence supports that SAI is a cholinergic-dependent marker of intra-cortical excitability. This neurophysiological technique has been already used in the context of pathophysiology understanding in both Parkinson’s disease and healthy aging. Previous works in PD suggested that central cholinergic activity is abnormal already in the early stages of disease, contributing to initial gait worsening under normal gait (Rochester et al., 2012) and to cognitive disturbances (Yarnall et al., 2013). Evidence in healthy aging, instead, linked central cholinergic activity to cognitive disturbances (Young-Bernier et al., 2012), but not yet to gait disturbances or falls.

Several previous studies support the “cholinergic hypothesis” of age-related cognitive decline (Bartus et al., 1982) by correlating imaging-derived measures of basal forebrain cholinergic structural decline with lower performance in older adults (Düzel et al., 2010; Hall et al., 2011; Wolf et al., 2014). In PD, the loss of basal forebrain cholinergic neurons and associated cortical cholinergic innervation is more extensive than in the brain of older adults and when patients with PD also develop dementia, cholinergic loss progresses most severely (Bohnen et al., 2003, 2006, 2012; Sarter et al., 2014). In addition, neuropathological studies have reported that almost 50% of the large cholinergic neurons of the pedunculopontine nucleus degenerate in PD (Pahapill and Lozano, 2000). Beyond showing cholinergic hypo-function both in aging and PD, experimental evidence on cholinergic decline in animal models and humans showed a close relationship between loss of cholinergic neurons and impairments in attention (Sarter and Bruno, 1998; Bohnen et al., 2006; Schliebs and Arendt, 2011).

The dual task paradigm is an optimal test for investigating the interplay between attention and motor control (O’Shea et al., 2002). The dual task deterioration of performance observed in our faller population, indicates that walking in our faller groups apparently required a heightened attentional control, likely because of a loss of automaticity. Indeed, a hallmark of normal control of walking is automaticity, which is the ability of the nervous system to successfully coordinate movement with minimal use of attention-demanding control resources (Clark, 2015). Performance of automated motor skills only requires minimal attentional demands, and needs little capacity resources, but if a motor skill is not automatic, it must utilize considerable attention and neural resources (Wu et al., 2015). In order to maintain normal walking movements, our faller population apparently tended to use attentional control strategies to bypass impaired automatic control, as evidenced by the sensitivity to dual tasking, and therefore, more attentional resources were needed. Perhaps this explains why attentional resources were not enough to sufficiently support the performance of the gait task properly during dual tasking. Here we showed that cholinergic dysfunction might be the neurophysiological substrate of a reduced ability to allocate attention, reduced gait monitoring during dual task and thus heightened risk of falls.

To explain the causal role of cholinergic hypo-function in aging and PD in inducing impairments in attention and thus increased risk of falls, an intriguing hypothesis has been suggested by Sarter et al. (2014). The authors proposed that when the loss of cortical cholinergic inputs impairs the attentional processing of gait, the striatal circuitry is “deprived” of this information, which it would normally use to select and sequence motor actions. In other words, dual cholinergic–dopaminergic loss attenuates the supervision of striatal circuitry and thereby “unmasks” the consequences of striatal dopaminergic denervation on gait (St. Peters et al., 2011).

There are some study limitations that should be acknowledged. First, cholinergic activity was evaluated at rest with sitting position. This is indeed a good and reliable method to measure cholinergic activity, as demonstrated by the fact that SAI is reduced or even abolished with administration of a selective muscarinic cholinergic receptor blocker in healthy participants (Di Lazzaro et al., 2000) and is reduced in Alzheimer’s disease (Di Lazzaro et al., 2002) and in other disorders characterized by cholinergic dysfunction (Di Lazzaro et al., 2007). However, it is worthwhile to address that recent studies showed that SAI is reduced in those muscles involved in a specific motor task during movement (Voller et al., 2006; Asmussen et al., 2013, 2014). In more details, decrease in SAI recorded during movement preparation was primarily mediated by supraspinal mechanisms, whereas both supraspinal and spinal mechanism contributed to SAI reduction during movement (Asmussen et al., 2013, 2014). These reductions in SAI when a muscle is involved in the task have been supposed to be necessary to allow somatosensory input to increase activity in the area of M1 responsible for the desired motor output (Asmussen et al., 2014).

In the present study we linked cholinergic dysfunction, as assessed by SAI, with motor performance deterioration under dual task (gait speed decrement). In this vein, it might be interesting in future studies to directly study whether an abnormal movement-induced modulation of SAI is present in fallers (either older adults and PD patients) and contributes to motor performance deterioration.

Second, in this study, we did not plan to test whether anticholinergic drugs may improve cholinergic activity as tested by SAI in our faller population. To date, a randomized, double-blind, cross-over trial is active in recruiting PD patients with the aim of examining the effects of augmentation of the cholinergic system on balance and gait (Mancini et al., 2015). Whether anticholinergic drugs may normalize cholinergic activity should be addressed in future study.

## Conclusion

Our findings suggest that central cholinergic activity may contribute to dual task gait disturbances, in older adults and PD fallers. Taking into account that dual task gait disturbance has been associated with fall risk, our findings give a strong rationale to use cholinergic therapy in the management of mobility problems in PD and in healthy aging, when fall risk is high.

## Author Contributions

EP, GA and LA contributed to the concept and rationale for the study. EP, CO, GL, GB and LA contributed to data collection. EP, CO, GL, GB, GA and LA contributed to data analysis. EP, AM, JMH, GA and LA contributed to the interpretation of the results and to the drafting of the manuscript. All authors participated in the approval of the final manuscript and take responsibility for the content and interpretation of this article.

## Funding

This work was supported by the European Commission, Grant number [FP7 project V-TIME-278169].

## Conflict of Interest Statement

The authors declare that the research was conducted in the absence of any commercial or financial relationships that could be construed as a potential conflict of interest.

## Abbreviations

MEPs: motor evoked potentials
MoCA: Montreal Cognitive Assessment
PD: Parkinson’s disease
SAI: short latency afferent inhibition
TMS: transcranial magnetic stimulation.
